# Association of Combat Experiences With Suicide Attempts Among Active-Duty US Service Members

**DOI:** 10.1001/jamanetworkopen.2020.36065

**Published:** 2021-02-02

**Authors:** Cynthia A. LeardMann, Rayna K. Matsuno, Edward J. Boyko, Teresa M. Powell, Mark A. Reger, Charles W. Hoge

**Affiliations:** 1 Deployment Health Research Department, Naval Health Research Center, San Diego, California; 2 Leidos, San Diego, California; 3 Seattle Epidemiologic Research and Information Center, Department of Veterans Affairs Puget Sound Health Care System, Seattle, Washington; 4 Department of Epidemiology, University of Washington School of Public Health, Seattle; 5 Department of Medicine, University of Washington School of Medicine, Seattle; 6 Veterans Affairs Puget Sound Healthcare System, Seattle, Washington; 7 Department of Psychiatry and Behavioral Sciences, University of Washington, Seattle; 8 Center for Psychiatry and Neuroscience, Walter Reed Army Institute of Research, Silver Spring, Maryland; 9 Psychiatry Division, Office of the Army Surgeon General, Falls Church, Virginia

## Abstract

**Importance:**

There is uncertainty about the role that military deployment experiences play in suicide-related outcomes. Most previous research has defined combat experiences broadly, and a limited number of cross-sectional studies have examined the association between specific combat exposure (eg, killing) and suicide-related outcomes.

**Objective:**

To prospectively examine combat exposures associated with suicide attempts among active-duty US service members while accounting for demographic, military-specific, and mental health factors.

**Design, Setting, and Participants:**

This cohort study analyzed data from the Millennium Cohort Study, an ongoing prospective longitudinal study of US service members from all military branches. Participants were enrolled in 4 phases from July 1, 2001, to April 4, 2013, and completed a self-administered survey at enrollment and every 3 to 5 years thereafter. The population for the present study was restricted to active-duty service members from the first 4 enrollment phases who deployed in support of the wars in Iraq and Afghanistan. Questionnaire data were linked with medical encounter data through September 30, 2015. Data analyses were conducted from January 10, 2017, to December 14, 2020.

**Exposures:**

Combat exposure was examined in 3 ways (any combat experience, overall combat severity, and 13 individual combat experiences) using a 13-item self-reported combat measure.

**Main Outcomes and Measures:**

Suicide attempts were identified from military electronic hospitalization and ambulatory medical encounter data using the *International Classification of Diseases, Ninth Revision* codes.

**Results:**

Among 57 841 participants, 44 062 were men (76.2%) and 42 095 were non-Hispanic White individuals (72.8%), and the mean (SD) age was 26.9 (5.3) years. During a mean (SD) follow-up period of 5.6 (4.0) years, 235 participants had a suicide attempt (0.4%). Combat exposure, defined broadly, was not associated with suicide attempts in Cox proportional hazards time-to-event regression models after adjustments for demographic and military-specific factors; high combat severity and certain individual combat experiences were associated with an increased risk for suicide attempts. However, these associations were mostly accounted for by mental disorders, especially posttraumatic stress disorder. After adjustment for mental disorders, combat experiences with significant association with suicide attempts included being attacked or ambushed (hazard ratio [HR], 1.55; 95% CI, 1.16-2.06), seeing dead bodies or human remains (HR, 1.34; 95% CI, 1.01-1.78), and being directly responsible for the death of a noncombatant (HR, 1.81; 95% CI, 1.04-3.16).

**Conclusions and Relevance:**

This study suggests that deployed service members who experience high levels of combat or are exposed to certain types of combat experiences (involving unexpected events or those that challenge moral or ethical norms) may be at an increased risk of a suicide attempt, either directly or mediated through a mental disorder.

## Key Points

**Question:**

What is the association of combat exposure with suicide attempts among active-duty US service members who were deployed in support of the wars in Iraq and Afghanistan?

**Findings:**

In this cohort study of 57 841 active-duty service members, high combat severity and certain combat experiences (ie, being attacked or ambushed, seeing dead bodies or human remains, and being directly responsible for the death of a noncombatant) were associated with suicide attempts. However, these associations were mostly accounted for by mental disorders, especially posttraumatic stress disorder.

**Meaning:**

Results of this study suggest that certain types of combat experiences may have different implications for service members than other experiences, increasing these individuals’ risk of attempting suicide, either directly or indirectly through a mental disorder.

## Teaser

This cohort study evaluates the association of specific combat exposures with suicide attempts among active-duty members of the US Armed Forces.

## Introduction

Suicide rates in the US military increased during the peak of the wars in Iraq and Afghanistan,^1,2^ prompting an examination of potential causes. This effort included studies that investigated the risk factors for suicides and suicide attempts, given the association of prior attempts with suicide deaths.^3,4,5,6,7^ One key theory was that the higher suicide rate was directly associated with deployments to Iraq or Afghanistan. However, the emerging body of research revealed the complex nature of this issue, demonstrating that although deployment is not associated with suicide, certain subgroups or specific combat experiences may increase risk.^8,9^ Previous research among service members has identified many suicide risk factors that are comparable to the risks among civilians, including mental disorders, behavioral factors, and relationship problems,^4,5,6,10,11,12^ but uncertainty remains about the potential role that certain aspects of military deployments may play in suicide-related outcomes.^9,13^ Of the priorities for additional research on deployment, the association of combat experiences with suicide-related outcomes is arguably among the most important. Despite public interest in and national commitment at the highest leadership levels to the topic,^14,15^ almost no research is available on the association of killing during combat, or other specific combat events, with subsequent suicide-related outcomes. Some studies have begun to examine the associations of combat exposure with suicide attempts and deaths.^9,13,16,17^ However, most of the previous research has defined combat exposure broadly.^5,12,13,18^ Although the findings are mixed and complicated by considerable heterogeneity in methods, a meta-analysis found no significant association of combat, defined broadly, with suicide attempts or deaths.^13^ Only a small number of cross-sectional studies have been conducted on the association between specific combat experiences (eg, killing) and suicide-related outcomes; these have been restricted to US veterans, National Guardsmen, and Canadian military personnel.^17,19^

Moreover, previous research has almost exclusively examined the direct association of combat with suicidal behaviors without considering the potential mediational relationships, particularly regarding mental disorders. Two cross-sectional studies suggested that posttraumatic stress disorder (PTSD) and depression symptoms mediate the association between combat and suicidal behaviors among veterans,^19,20^ whereas a study among deployed Air Force personnel found no direct or indirect role for PTSD in explaining the association between combat and suicidal behaviors.^16^ Given that existing research is limited and has inconsistent results, more investigation is needed for a better understanding of the association of combat exposure with suicide attempts among US military service members who were deployed.

Using linked Millennium Cohort Study and medical encounter data, we conducted this study with the aim of prospectively examining combat exposures associated with suicide attempts in a large population of active-duty service members across all branches of the US military while accounting for demographic, military-specific, and mental health factors.

## Methods

### Population and Data Sources

The Millennium Cohort Study is an ongoing longitudinal study that examines the long-term health effects of military service; details of the study methods have been previously described.^21,22,23^ Currently, the Millennium Cohort Study consists of more than 200 000 participants who enrolled in 4 phases between July 1, 2001, and April 4, 2013, representing all service branches and components (ie, active duty, reserves, National Guard). Participants completed a self-administered survey at enrollment and approximately every 3 to 5 years thereafter. The survey questionnaires encompassed a wide range of topics, including physical, mental, and behavioral health as well as military and nonmilitary life experiences. Participants provided voluntary, informed consent, including permission to link their data to US Department of Defense records such as medical encounters. This study was approved by the Naval Health Research Center Institutional Review Board. We followed the Strengthening the Reporting of Observational Studies in Epidemiology (STROBE) reporting guideline.

The population for this cohort study was restricted to active-duty service members from the first 4 enrollment phases who had deployed in support of the military operations in Iraq and Afghanistan (n = 85 768). To examine the factors associated with incident suicide attempts, we excluded individuals with a suicide attempt before enrollment or first deployment (n = 230). Eligible participants must have completed a survey at enrollment and at least 1 of the questionnaires that included the combat-specific experiences (ie, 2007-2008 or 2011-2013) (n = 61 031). After exclusion of participants with missing data at enrollment (ie, demographic, military-specific, or mental health factors), the final study population consisted of 57 841 participants.

Demographic and military-specific data (sex, birth date, race/ethnicity, deployment dates, pay grade, service component, and service branch) were obtained from electronic personnel files maintained by the Defense Manpower Data Center. Educational attainment and marital status were assessed using self-reported enrollment data. Medical encounter data were obtained from the Department of Defense Military Health System Data Repository.

### Suicide Attempts and Combat Experiences

Suicide attempts were ascertained from military electronic hospitalization (inpatient) and ambulatory (outpatient) medical encounter data using *International Classification of Diseases, Ninth Revision* (*ICD-9*) E950-E958 codes between October 1, 2000, to September 30, 2015. The data reflected standardized diagnoses within the Military Health System, including care covered by TRICARE insurance but received outside of military treatment facilities. Incident suicide attempts were assessed on the basis of the earliest medical encounter with a suicide attempt *ICD-9* code between enrollment and September 30, 2015.

Combat was assessed (on the 2007-2008 and/or 2011-2013 survey) using a 13-item combat measure that captured experiences encountered during deployments in support of the conflicts in Iraq and Afghanistan from 2001 to the end of the follow-up period.^24,25^ For each item, responses to combat experiences (eg, being attacked or ambushed; being wounded or injured) were dichotomized. Combat exposure was examined in 3 ways: any combat experience (0 vs ≥1 experience), overall combat severity, and 13 individual combat experiences. Combat severity was based on the total types of combat experiences: no combat (0 items), low combat (1-3 items), medium combat (4-7 items), and high combat (8-13 items).

### Mental Health

Posttraumatic stress disorder was assessed using the 17-item PTSD Patient Checklist Civilian Version (PCL-C) and was defined as reporting a moderate or higher level of at least 1 intrusion, 3 avoidance, and 2 hyperarousal symptoms, according to criteria established by the *Diagnostic and Statistical Manual of Mental Disorders, Fourth Edition* (*DSM-IV*).^26^ Participants were classified as having PTSD if they had a positive screening result for PTSD or if they reported ever being diagnosed with PTSD by a health professional.^5^

The 8 items of the Patient Health Questionnaire (PHQ-8) were used to screen for depression using the depression diagnosis scoring algorithm from *DSM-IV*.^27,28^ Participants were classified as having depression if they had a positive screening result or if they reported ever being diagnosed with depression by a health professional.^5^ The presence of PTSD or depression was assessed at enrollment and on follow-up surveys. Posttraumatic stress disorder and depression were combined to create a 4-level mental health variable (neither PTSD nor depression, PTSD only, depression only, and comorbid PTSD and depression).

### Statistical Analysis

Descriptive analyses compared demographic, military-specific, and mental health factors and combat exposure by suicide attempts. Hazard ratios (HRs) with 95% CIs were estimated with Cox proportional hazards time-to-event regression models. Nested models were conducted to estimate the association between each of the 15 combat exposures (ie, any combat experience, overall combat severity, and 13 individual combat experiences) and suicide attempts, as follows: unadjusted (model 1); adjusted for demographic and military-specific factors (model 2); and adjusted for demographic, military-specific, and mental health factors (model 3). All adjustment variables were assessed at the time of enrollment. Interactions of mental health with combat exposures for suicide attempts were tested in model 3, in which a 2-sided *P* < .05 was considered statistically significant. Person-years for each participant were calculated from the time of enrollment to the date of (1) first suicide attempt, (2) separation from the military, (3) change to reserve component status, (4) study completion (September 30, 2015), or (5) death, whichever occurred first. Schoenfeld residuals indicated that proportional hazards assumptions were met in the Cox models, and the final multivariable models did not indicate multicollinearity (defined as a variance inflation factor of ≥4).

Given the attenuation of HRs when the mental health factor was added, we examined whether the association between combat severity and suicide attempt was mediated by postdeployment PTSD or depression (Figure). To ensure accurate temporal assessment, we excluded individuals with missing postdeployment PTSD and depression data (n = 2170) from these analyses (n = 55 671). We dichotomized the mediator (PTSD or depression vs no PTSD and depression) to use established formulas and a SAS macro (version 9.4; SAS Institute), because suitable methods for examining multilevel mediators in survival analyses are limited.^29,30^ Using these formulas, we estimated the direct (holding PTSD or depression constant) and indirect (mediated through PTSD or depression) association of high combat severity with suicide attempt, and the proportion mediated, while adjusting for demographic and military-specific factors. To further examine the specific outcome of PTSD compared with depression, we conducted a *z* test using the standard coefficients using a 4-level mediator (neither, PTSD only, depression only, and comorbid PTSD and depression).^31^

**Figure.**
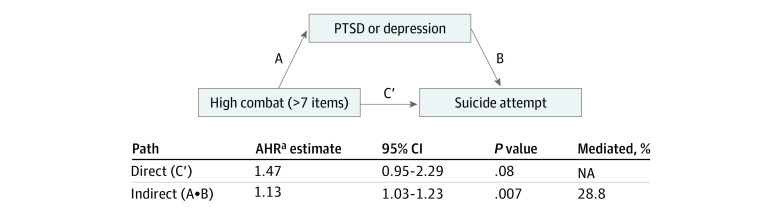
Mediation Analyses Examining Postdeployment Posttraumatic Stress Disorder (PTSD) and/or Depression Between High Combat Severity and Suicide Attempt AHR indicates adjusted hazard ratio. ^a^Adjusted for sex, race/ethnicity, age, marital status, educational attainment, service branch, and pay grade.

Data management and statistical analyses were performed using SAS software. Work on these analyses was conducted from January 10, 2017, to December 14, 2020.

## Results

The study population was composed of 57 841 active-duty service members, who were predominantly men (44 062 [76.2%]) with a mean (SD) age of 26.9 (5.3) years, had a non-Hispanic White race/ethnicity (42 095 [72.8%]), had less than a Bachelor's degree educational attainment (43 492 [75.2%]), had a junior enlisted pay grade (42 298 [73.1%]), and were in the Army (23 824 [41.2%]). During a mean (SD) follow-up of 5.6 (4.0) years, 235 participants were documented as having a nonfatal suicide attempt (72.51 suicide attempts per 100 000 person-years). The demographic, military-specific, and mental health factors were statistically significantly associated with suicide attempts, with the exception of race/ethnicity and marital status (Table 1). Compared with those without a suicide attempt, participants with a suicide attempt were proportionally more likely to endorse each of the 13 individual combat experiences and report 4 or more types of combat experiences (Table 2).

**Table 1.** Characteristics of Study Participants Stratified by Suicide Attempt

Characteristic^a^	No. (%)	
	Without suicide attempt (n = 57 606)^b^	With suicide attempt (n = 235)
Demographic		
Age, mean (SD), y	26.9 (5.3)	25.3 (4.6)
Sex		
Male	43 901 (76.2)	161 (68.5)
Female	13 705 (23.8)	74 (31.5)
Race/ethnicity		
White, non-Hispanic	41 936 (72.8)	159 (67.7)
Black, non-Hispanic	6521 (11.3)	30 (12.8)
Other^c^	9149 (15.9)	46 (19.6)
Marital status		
Single, never married	19 371 (33.6)	75 (31.9)
Married	32 798 (56.9)	130 (55.3)
Divorced, separated, widowed	5437 (9.4)	30 (12.8)
Educational attainment		
<Bachelor’s degree	43 278 (75.1)	214 (91.1)
≥Bachelor's degree	14 328 (24.9)	21 (8.9)
Military-specific		
Service branch		
Army	23 688 (41.1)	136 (57.9)
Navy/Coast Guard	10 099 (17.5)	29 (12.3)
Marine Corps	6297 (10.9)	17 (7.2)
Air Force	17 522 (30.4)	53 (22.6)
Military pay grade		
Junior enlisted (E01-E05)	42 084 (73.1)	214 (91.1)
Senior enlisted or officer	15 522 (27.0)	21 (8.9)
Mental health		
PTSD and/or depression		
Neither	49 493 (85.9)	150 (63.8)
PTSD only^d^	2242 (3.9)	17 (7.2)
Depression only^e^	2885 (5.0)	31 (13.2)
Comorbid PTSD and depression^d^^,^^e^	2986 (5.2)	37 (15.7)

Abbreviation: PTSD, posttraumatic stress disorder.

^a^ All characteristics were assessed at the time of enrollment.

^b^ All characteristics, except race/ethnicity and marital status, were statistically significant at the *P* < .05 level.

^c^ Includes individuals who identify as American Indian, Asian, Pacific Islanders, Hispanic/Latino, mixed race/ethnicity, or another race/ethnicity not considered non-Hispanic White or non-Hispanic Black.

^d^ PTSD was assessed using the 17-item PTSD Patient Checklist Civilian Version defined by the *Diagnostic and Statistical Manual of Mental Disorders, Fourth Edition* criteria and/or an affirmative response to a provider diagnosis.

^e^ Assessed using 8 items in the Patient Health Questionnaire and/or an affirmative response to a provider diagnosis.

**Table 2.** Frequencies of Combat Exposures by Suicide Attempt Among Study Participants Who Deployed (n = 57 841)

Combat exposure	No. (%)	
	Without suicide attempt (n = 57 606)	With suicide attempt (n = 235)
Overall		
Any combat experience		
No	14 162 (24.6)	46 (19.6)
Yes	43 444 (75.4)	189 (80.4)
Combat severity		
No combat: 0 items	14 162 (24.6)	46 (19.6)
Low combat: 1-3 items	16 420 (28.5)	49 (20.9)
Medium combat: 4-7 items	14 544 (25.3)	61 (26.0)
High combat: 8-13 items	12 480 (21.7)	79 (33.6)
Individual combat items		
Feeling in great danger of being killed	32 768 (56.9)	153 (65.1)
Being attacked or ambushed	27 699 (48.1)	147 (62.6)
Receiving small arms fire	23 948 (41.6)	120 (51.1)
Clearing/searching homes or buildings	12 044 (20.9)	70 (29.8)
Having an IED or a booby trap explode near you	15 728 (27.3)	94 (40.0)
Being wounded or injured	6476 (11.2)	37 (15.7)
Seeing dead bodies or human remains	22 419 (38.9)	119 (50.6)
Handling or uncovering human remains	10 536 (18.3)	61 (26.0)
Knowing someone seriously injured or killed	27 923 (48.5)	137 (58.3)
Seeing Americans seriously injured or killed	23 552 (40.9)	118 (50.2)
Having a member of your unit seriously injured or killed	21 816 (37.9)	111 (47.2)
Being directly responsible for the death of an enemy combatant	6931 (12.0)	44 (18.7)
Being directly responsible for the death of a noncombatant	1453 (2.5)	14 (6.0)

Abbreviation: IED, improvised explosive device.

Unadjusted proportional hazards models revealed that experiencing any combat, high combat severity, and the 13 individual combat experiences were significantly associated with suicide attempts (Table 3). After adjustments for demographic and military-specific factors, high combat severity and 7 of the individual combat experiences remained statistically significant (Table 3). Once adjusted for mental disorders, however, only 3 individual combat experiences remained significant: being attacked or ambushed (HR, 1.55; 95% CI, 1.16-2.06), seeing dead bodies or human remains (HR, 1.34; 95% CI, 1.01-1.78), and being directly responsible for the death of a noncombatant (HR, 1.81; 95% CI, 1.04-3.16). A statistically significant interaction was found between mental disorders and being directly responsible for the death of an enemy combatant with risk of suicide attempts. Models stratified by mental health status (neither, PTSD only, depression only, comorbid PTSD and depression) indicated that among those with depression only, being directly responsible for the death of an enemy combatant was significantly associated with an increased risk of suicide attempts (adjusted HR, 5.85; 95% CI, 2.26-15.17). No other interaction terms were found to be statistically significant.

**Table 3.** Effect Estimates of Suicide Attempts by Combat Exposure Among Active-Duty Service Members Who Deployed in Support of the Operations in Iraq and Afghanistan (n = 57 841)

Combat exposure^a^	Model 1: Unadjusted, HR (95% CI)	Adjusted HR (95% CI)	
		Model 2: Adjusted for demographic and military-specific factors^b^	Model 3: Adjusted for demographic, military-specific, and mental health factors^c^
Overall			
Any combat experience			
No	1 [Reference]	1 [Reference]	1 [Reference]
Yes	1.39 (1.01-1.92)^d^	1.14 (0.81-1.62)	1.02 (0.72-1.44)
Combat severity			
No combat: 0 items	1 [Reference]	1 [Reference]	1 [Reference]
Low combat: 1-3 items	0.92 (0.62-1.38)	0.89 (0.59-1.34)	0.84 (0.56-1.26)
Medium combat: 4-7 items	1.37 (0.93-2.00)	1.22 (0.81-1.84)	1.09 (0.72-1.64)
High combat: 8-13 items	2.11 (1.46-3.04)^d^	1.77 (1.15-2.72)^d^	1.41 (0.91-2.18)
Individual combat items			
Feeling in great danger of being killed	1.51 (1.16-1.98)^d^	1.24 (0.93-1.66)	1.08 (0.81-1.46)
Being attacked or ambushed	1.90 (1.46-2.48)^d^	1.71 (1.29-2.27)^d^	1.55 (1.16-2.06)^d^
Receiving small arms fire	1.54 (1.19-1.99)^d^	1.30 (0.98-1.72)	1.17 (0.88-1.56)
Clearing/searching homes or buildings	1.69 (1.28-2.23)^d^	1.46 (1.07-1.99)^d^	1.32 (0.97-1.81)
Having an IED or a booby trap explode near you	1.83 (1.41-2.37)^d^	1.50 (1.13-2.00)^d^	1.33 (0.99-1.78)
Being wounded or injured	1.61 (1.14-2.29)^d^	1.33 (0.93-1.90)	1.10 (0.77-1.59)
Seeing dead bodies or human remains	1.69 (1.31-2.18)^d^	1.51 (1.14-2.00)^d^	1.34 (1.01-1.78)^d^
Handling or uncovering human remains	1.59 (1.19-2.13)^d^	1.35 (0.99-1.84)	1.17 (0.86-1.61)
Knowing someone seriously injured or killed	1.58 (1.22-2.05)^d^	1.31 (0.98-1.76)	1.17 (0.87-1.57)
Seeing Americans seriously injured or killed	1.52 (1.17-1.96)^d^	1.35 (1.03-1.78)^d^	1.20 (0.90-1.58)
Having a member of your unit seriously injured or killed	1.55 (1.20-2.00)^d^	1.22 (0.91-1.63)	1.09 (0.81-1.46)
Being directly responsible for the death of an enemy combatant	1.76 (1.26-2.44)^d^	1.60 (1.12-2.27)^d^	1.38 (0.96-1.98)^e^
Being directly responsible for the death of a noncombatant	2.68 (1.56-4.61)^d^	2.33 (1.34-4.03)^d^	1.81 (1.04-3.16)^d^

Abbreviations: HR, hazard ratio; IED, improvised explosive device; PTSD, posttraumatic stress disorder.

^a^ Each combat exposure was run in a separate model.

^b^ A separate model was conducted for each combat exposure as the main exposure, adjusted for age, sex, race/ethnicity, marital status, educational attainment, service branch, and pay grade.

^c^ A separate model was conducted for each combat exposure as the main exposure, adjusted for age, sex, race/ethnicity, marital status, educational attainment, service branch, pay grade, and PTSD and/or depression.

^d^ *P* < .05.

^e^ Mental health significantly moderated the association of being directly responsible for the death of an enemy combatant with a suicide attempt; therefore, the model was stratified by mental health status. Of the stratified groups, only 1 indicated that being directly responsible for the death of an enemy combatant was significantly associated with increased risk of suicide attempt. Among those with depression only, being directly responsible for the death of an enemy combatant significantly increased the risk of a suicide attempt (adjusted HR, 5.85; 95% CI, 2.26-15.17).

In the mediation analyses, postdeployment PTSD or depression significantly mediated the association of high combat severity with suicide attempts (HR, 1.13; 95% CI, 1.03-1.23; *P* = .007) (Figure). An estimated 28.8% of the association was mediated through PTSD or depression. The additional analysis demonstrated that combat severity exhibited a significant indirect association with suicide attempts through PTSD. Specifically, high combat exposure was associated with PTSD only (β = 2.07; *P* < .001) and comorbid PTSD and depression (β = 2.01; *P* < .001), which, in turn, were associated with suicide attempts (β = 0.66 [*P* = .003] for PTSD only, and β = 0.72 [*P* < .001] for comorbid PTSD and depression). However, postdeployment depression without PTSD did not significantly mediate the association between combat severity and suicide attempts.

In addition to the suicide attempts, 26 participants were censored in the analysis because of death by suicide during the follow-up period. When these individuals were reclassified as having an attempt (defining the outcome as a nonfatal or fatal suicide attempt), the results were generally consistent with those from the nonfatal suicide attempt analyses. In the final adjusted analyses (model 3), having an improvised explosive device or booby trap explode near you was significantly associated with a nonfatal or fatal suicide attempt (HR, 1.34; 95% CI, 1.01-1.77), whereas the risk associated with being directly responsible for the death of a noncombatant remained elevated but was no longer significant (HR, 1.64; 95% CI, 0.94-2.85). The other estimates from model 3 and mediation results remained consistent.

## Discussion

This study expands on previous research that highlighted the complex association between combat and suicide-related outcomes. To our knowledge, this study is one of the few to examine specific experiences of combat associated with the risk of suicide attempts among active-duty military personnel who were deployed in support of the operations in Iraq and Afghanistan. The results can be summarized into 4 main findings.

First, combat, when defined broadly, was not associated with suicide attempts after adjustments for demographic and military-specific factors. Second, high combat severity was associated with an increased risk for suicide attempts, although this association was partly mediated by mental disorders, specifically PTSD. Third, although some individual combat experiences were associated with suicide attempts, these associations were largely accounted for by mental disorders. Only 3 individual combat experiences were significantly associated with suicide attempts after adjusting for mental disorders, including being attacked or ambushed, seeing dead bodies or human remains, and being directly responsible for the death of a noncombatant. Fourth, the associations between exposure to being directly responsible for the death of an enemy combatant and a suicide attempt varied by the presence of depression, whereby being directly responsible for the death of an enemy combatant was associated with a significantly increased risk for suicide attempts only among those with depression.

The lack of a significant association between combat, as an any combat experience, and suicide attempts was consistent with findings from a previous meta-analysis^13^ and is an important finding for understanding differences in results across studies based on how combat has been assessed. This null finding may be explained by combining service members with different risk profiles with varying levels of combat experiences. After we categorized individuals according to the total types of combat exposure, we found that service members who reported the greatest number of combat experiences had an elevated risk of suicide attempts. Consistent with findings from a study of Canadian Armed Forces, the magnitude and significance of this association decreased after adjustment for demographic and military-specific factors, and the association was no longer significant after an adjustment for mental disorders.^17^ This risk attenuation suggests a nuanced association. It has been well established that combat is associated with increased risk of postdeployment mental disorders and that mental disorders are associated with increased risk for suicidal behaviors, but only a limited number of studies have examined this indirect association. Findings from this study aligned with the results of 2 previous studies that suggested that those who experienced combat had an increased risk of developing PTSD, which in turn may elevate their risk for attempting suicide.^19,20^ The results, however, differed from those of a study of deployed Air Force personnel, in which no direct or indirect association was found between combat experiences and self-reported suicidal behaviors.^16^ Methodological differences between studies may explain these seemingly conflicting findings.

Findings from this study also suggest that some specific combat experiences have a stronger association with suicide attempts than others. Of the 13 individual combat experiences, 7 were associated with suicide attempts after adjusting for demographic and military-specific factors, and 3 remained significant after accounting for mental health at enrollment. These findings partly align with those from a study of Canadian Armed Forces, in which only 1 of the 12 deployment-related traumatic events (life-threatening situation and unable to respond because of rules of engagement) was associated with attempting suicide after adjusting for mental health.^17^

Although it is difficult to know why certain experiences have a stronger association with attempting suicide than other experiences, clinicians may want to consider factors, such as the sudden unexpected nature of certain events and feelings of helplessness or loss of control (eg, being attacked or ambushed; seeing dead bodies or human remains), compared with more active engagements, such as clearing or searching homes or buildings. Repetitive exposure to death over time may also be associated with an acquired capability for suicide.^32^ The risk of attempting suicide remained elevated for those who reported being directly responsible for the death of a noncombatant after adjusting for mental health status. The inadvertent killing of a noncombatant can be particularly challenging to moral and ethical norms, and other research has shown that killing a noncombatant may have a stronger and more consistent association with mental health outcomes than being directly responsible for the death of an enemy combatant.^33^ Although it is not clear why exposure to being directly responsible for the death of an enemy combatant was associated with attempting suicide only among those with depression, it may be associated with a multiplicative effect of the killing combined with depression. Although being responsible for the death of an enemy combatant is a potential military occupational experience for which service members train, it may have greater adverse effects for those with depression than those without depression. After we redefined the outcome (nonfatal or fatal suicide attempts), we found some slight differences between which individual combat experiences were associated with the outcome. These findings highlight the importance of distinguishing the questions associated with the subject of killing during wartime operations and the need for more in-depth research into the differences between fatal and nonfatal suicide attempts.

### Strengths and Limitations

A key strength of this study is that it is the largest longitudinal US military study, to our knowledge, to evaluate the associations between combat experiences and suicide attempts during a time when combat deployment occurred with high frequency. The survey instrument prospectively collected extensive information, which cannot be accessed elsewhere. Because individuals with mental health symptoms may not seek treatment,^25^ the PCL-C and PHQ-8 instruments may more thoroughly and completely capture service members with mental health symptoms.

This study has several limitations. It relied on administrative data systems for identifying suicide attempts. The use of *ICD-9* codes to identify suicide attempts may have underestimated the true number of suicide attempts given that attempts may not have been associated with a health care visit or specific *ICD-9* diagnostic code, or participants may have sought care elsewhere. Although underreporting might limit statistical power, it should not detract from the meaningful associations observed. As with any prospective cohort study, in this study loss to follow-up was a concern. However, the outcome of interest relied on medical records. Investigations have found the Millennium Cohort to be representative with reliable reporting of data.^22,34,35^ Although the PCL-C and PHQ-8 instruments are standardized and validated, they are surrogates for a clinical diagnosis. In addition, it is not possible to know with certainty when these conditions first occurred compared with deployment and the outcomes of interest; however, the associations showed consistency across variables and analytic approaches.

## Conclusions

Results of this cohort study suggest that being attacked or ambushed, seeing dead bodies or human remains, and being directly responsible for the death of a noncombatant may have different implications for service members than other combat experiences, increasing the risk of a suicide attempt. Thus, clinicians should be aware of the importance of inquiring about the nature of combat experiences, paying particular attention to experiences that may involve unexpected events, more passive events that may be associated with feelings of helplessness, or events that are more challenging from a moral or an ethical perspective. The results also suggest that combat experiences may be indirectly associated with suicide attempt risk through PTSD. Hence, these findings emphasize the continued importance of identification and treatment of PTSD and comorbid PTSD and depression.
